# Diabetes reprograms brain and systemic immune landscapes to impair repair after injury

**DOI:** 10.1186/s13041-026-01310-5

**Published:** 2026-06-09

**Authors:** Keon Ah Lee, Su Yeon Kim, Jin Soo Lee, Haijie Yang, Jiawei An, Sang Myun Park, Eun Jeong Lee, Eun-Hye Joe

**Affiliations:** 1 Center for Convergence Research of Neurological Disorders, Ajou University School of Medicine, Suwon, 16499 Kyunggi-do Korea; 2 Department of Pharmacology, Ajou University School of Medicine, Suwon, 16499 Kyunggi-do Korea; 3 Neuroscience Graduate Program, Department of Biomedical Sciences, Ajou University School of Medicine, Suwon, 16499 Kyunggi-do Korea; 4 Department of Brain Science, Ajou University School of Medicine, Suwon, 16499 Kyunggi-do Korea; 5 Department of Neurology, Ajou University School of Medicine, Suwon, 16499 Kyunggi-do Korea

## Abstract

Diabetes is a major risk factor for poor outcomes following brain injury, yet the underlying mechanisms remain incompletely understood. Using stereotaxic ATP injection into the striatum combined with 9.4T MRI, we found that diabetic mice exhibited larger initial lesions and delayed recovery compared to controls, even after matching for initial lesion size. Transcriptomic profiling of the intact diabetic brain revealed extensive gene expression changes before injury, with prominent downregulation of excitatory synaptic transmission and cytoskeletal organization pathways. RNA-seq of peripheral blood mononuclear cells (PBMCs) showed that diabetes altered systemic immune response, mitochondrial metabolism, and cell structure–related genes after injury. Infiltrating monocytes in diabetic brains exhibited dispersed distribution, and transcriptional remodeling characterized by upregulation of transporters and channels and downregulation of mitochondrial and prostaglandin biosynthesis genes. Furthermore, diabetes attenuated astrocyte activation, impairing monocyte distribution. These results reveal that diabetes induces pre-injury transcriptional reprogramming in both the brain and immune system, leading to heightened injury severity and impaired repair responses.

**Supplementary Information:**

The online version contains supplementary material available at 10.1186/s13041-026-01310-5.

## Introduction

Neurodegenerative diseases have been studied extensively at the point of the death of neurons. However, insufficient repair of brain injury may result in accumulation of damage, playing a role in the onset and/or progression of neurodegenerative diseases [1, 2]. In injured brain, not only brain cells including neurons and glia but blood inflammatory cells cooperate to protect brain cells from further injury and to repair the injury [3–6]. Microglia isolate injury sites to prevent further injury [7–9], and astrocytes are activated to produce growth factors [10, 11], and express chemokines to recruit monocytes from blood [4, 12]. Infiltrated monocytes play a role in the repair of injured brains through several ways: monocytes phagocyte dead/dying cells [4, 5, 13–15], and produce exosomes through pyroptosis, which induces neurite outgrowth and astrocyte process elongation toward damage core [4, 6, 16, 17]. Monocytes also induce neurogenesis and angiogenesis, and recruit and/or promote the proliferation of astrocytes and oligodendrocytes [4, 17, 18]. Therefore, changes in the function of monocytes as well as resident brain cells may substantially alter repair processes.

Diabetes increases the risk of neurodegenerative diseases including Alzheimer’s disease (AD) and Parkinson’s disease (PD) [18–22]. It has been reported that diabetes impairs the conversion of pro-inflammatory Ly6C^hi^ monocytes into reparative Ly6C^lo^ monocytes, a transition that is critical for efficient tissue repair [23]. Diabetes also increases the number of circulating monocytes, thereby contributing to a heightened pro-inflammatory milieu [24, 25]. Single-cell RNA sequencing of human diabetic foot ulcer tissues further revealed that monocytes from diabetic patients exhibit elevated expression of pro-inflammatory mediators such as TNF-α, IL-1β, and CCL2, while showing reduced expression of genes associated with tissue repair and angiogenesis, including VEGFA and IGF1 [26–28]. In parallel, peripheral blood mononuclear cells (PBMCs) from diabetic patients show suppressed activation of key immune and inflammatory pathways, including type I interferon signaling and TLR-mediated responses [26]. Moreover, diabetes diminishes the phagocytic capacity of monocytes, limiting their ability to clear apoptotic cells and debris [27]. Interestingly, recent findings showed that Galectin-3–positive (Gal3⁺) monocytes in the diabetic brain paradoxically delay microvascular repair, as these cells engage in excessive phagocytosis of blood vessels, thereby hindering vascular regeneration [29]. Taken together, these findings indicate that diabetes not only alters monocyte numbers but also disrupts their reparative functions, which may critically contribute to impaired recovery following brain injury.

In this study, we investigated the underlying mechanisms by which diabetes increases damage and delays recovery using a streptozotocin (STZ)-induced diabetic mouse model. We found that STZ altered gene expression even in intact brains, which may increase susceptibility to initial injury. In response to injury, STZ delayed repair, which may be associated with impaired astrocyte activation, and spatially dispersed monocyte infiltration although increased expression of repair-related genes. These findings suggest that diabetes-induced changes in both resident glial cells and infiltrating monocytes underlie the impaired recovery of brain injury.

## Materials and methods

### Ethical approval and to participate

All experiments were performed in accordance with approved animal protocols and guidelines established by the Ajou University School of Medicine Ethics Review Committee for animal experiments, and all animal work was approved by the Ethical Committee for Animal Research of Ajou University (2022—0043; AMC119).

### Animals

Male mice (C57BL/6 J, 8 weeks old, 23–25 g) were housed under a 12 h light/dark cycle with free access to food and water.

### Development of the diabetic mouse model

To create the STZ (S0130, Sigma) mouse model, we referenced the following papers [30]. Briefly, hyperglycemia was induced by the administration of multiple, low-doses of STZ (50 mg/kg, intraperitoneally; i.p.) to mice on 5 consecutive days (SFig. 1a). STZ powder was dissolved in citrate buffer (pH 4.5), stored at 4℃ until use, and all injections were made within 20 min of dissolution. Blood glucose was measured 13 days after the last injection using a blood glucose meter (Accu-chek Performa, Roche, USA). We considered hyperglycemia to be induced if the blood glucose level was > 300 to 600 mg/dl. Diabetic states were confirmed by decreased body weights and increased fasting glucose levels (SFig. 1b, c).

### Stereotaxic injection

Mice were anesthetized by intraperitoneal injection of tribromoethanol (250 mg/kg; Sigma, St. Louis, MO) and placed in a stereotaxic apparatus (David Kopf Instruments, Tujunga, CA). Brain injury was produced by unilateral administration of 400 nmol of ATP (A2383, Sigma) into the striatum (AP, + 1.0 mm; ML, − 1.9 mm; DV, − 3.5 mm from bregma) (SFig. 1a), according to stereotaxic coordinates from the Atlas of the Mouse Brain (Paxinos and Franklin, 2nd edition), as previously described [31]. ATP is a component of damage-associated molecular patterns produced under pathological conditions that induce acute brain damage through activation of ionotropic purinergic receptors, P2X7 [31–34]. A Hamilton syringe (701RN, 10ul, 26 s/2"/2) was attached to a syringe pump (KD Scientific, New Hope, PA) and equipped with a 33-gauge needle (small Hub RN, 1 inch, 45°, 33GA RN 6PK, point style 4). ATP was dissolved in sterile phosphate-buffered saline (PBS), and 0.8 μl of ATP solution was infused at a rate of 0.2 μl/min.

### MR image analysis

Mice were anesthetized with isoflurane at the indicated times after ATP injection using 9.4 T MRI (BioSpec 94/30 US/R, Bruker, Billerica, MA) and a volume RF coil (inner diameter, 23 mm, Bruker) (Sungkyunkwan University, Korea). T2-weighted 2D Turbo RARE sequences were acquired using the following parameters: TR/TE, 9,000/33 ms; resolution, 78 × 78 × 250 μm^3^; slice thickness, 250 μm; RARE factor, 8; average, 2; scan time, 9 min 36 s. Lesion volumes were quantified from MR images using ImageJ.

### Tissue preparation

Mice were anesthetized and first transcardially perfused with PBS containing 0.5% sodium nitrate and heparin (10 U/ml), followed by 4% paraformaldehyde (PFA) in 0.1 M phosphate buffer (PB; pH 7.2). Brains were placed in 4% paraformaldehyde at 4℃ for 1 day, and then kept in 30% sucrose in PBS until they sank. For immunostaining, coronal sections (35 μm) were obtained using a cryostat (Leica, Wetzlar, Germany) and stored in anti-freeze stock solution (PB containing 30% glycerol and 30% ethylene glycol, pH 7.2) at 4℃ before use. For protein and mRNA preparation, brains were removed after transcardial perfusion with cold sterile PBS, and tissue blocks (2 × 2 × 2 mm^3^) were cut using an Alto mouse brain slicer matrix (Roboz Surgical Instruments, Gaithersburg, MD) and a razor blade, and stored at − 70℃until use.

### Isolation of brain-infiltrating monocytes

From brain tissues, single-cell suspensions were prepared using the Adult Brain Dissociation Kit (Miltenyi Biotec, Bergisch Gladbach, Germany) according to the manufacturer’s instructions. Cells were stained with a fluorescein isothiocyanate (FITC)-conjugated anti-CD45 antibodies, and CD45⁺ cells were isolated with a FACSAria III cell sorter (BD Biosciences, San Jose, CA, USA). The sorted cell populations were subsequently processed for whole-transcriptome sequencing.

### Isolation of peripheral blood mononuclear cells (PBMCs)

Mice were anesthetized, and blood was collected via cardiac puncture into heparin-coated tubes. After dilution with PBS (1:1), blood was carefully layered onto Ficoll-Paque PLUS (Cytiva, Uppsala, Sweden), and centrifuged at 400 × g for 30 min at room temperature without brake. The mononuclear cell layer (PBMCs) at the plasma–Ficoll interface was carefully collected. The cells were washed twice with PBS at 300 × g for 10 min. PBMCs were immediately lysed with TRI Reagent (MRC, Inc., Cincinnati, OH) and processed for whole-transcriptome sequencing.

### RNA sequencing and data analysis

Total RNA was extracted from brain tissues or cells using the Tri reagent (MRC, Inc., Cincinnati, OH). Illumina-compatible libraries were constructed using a TruSeq RNA library preparation kit (Illumina, CA, USA), according to the manufacturer's instructions. In brief, mRNA was purified from total RNA by polyA selection and was then chemically fragmented and converted into single-stranded cDNA using random hexamer priming. RNA libraries were constructed by PCR amplification and quantified by quantitative PCR (qPCR) using an Agilent 2100 Bioanalyzer, according to the qPCR Quantification Protocol Guide (Agilent Technologies Inc., San Diego, CA). The cDNA library was sequenced using the HiSeq2000 platform (Illumina). Sequence analyses were performed by Macrogen (Seoul, Korea). Sequence quality was verified using FastQC v0.10.0. Raw data were log2 transformed and analyzed using RStudio software. Functions enriched in gene sets were analyzed using gene ontology (GO) categories.

#### Differential expression analysis

Differential expression analysis was performed using the NOISeq R package. For each time point (Day 0, 3, 7, and 14), tissue/RNA from at least four mice was pooled to generate one pooled sample per time point (≥ 4 mice/time point; n = 1 pooled sample per time point). Because biological replicates were not available at each time point, NOISeq was used to estimate the noise distribution by simulating technical replicates and to identify differentially expressed genes (DEGs). DEGs were defined using a probability threshold of q ≥ 0.8, which corresponds to an odds of approximately 4:1 that a gene is truly differentially expressed rather than noise, as described in the NOISeq framework [35].

#### Western blot analysis

Tissues were homogenized in a modified RIPA buffer (10 mM sodium phosphate buffer pH 7.2, 150 mM NaCl, 1% NP-40, 0.5% sodium deoxycholate) containing phosphatase and protease inhibitor cocktails (GenDEPOT, Barker, TX). Proteins were separated by sodium dodecyl sulfate–polyacrylamide gel electrophoresis (SDS-PAGE) and transferred to a nitrocellulose membrane (GE Healthcare, Pittsburgh, PA). After blocking with 5% skim milk, the membranes were incubated overnight at 4 °C with primary antibodies: rabbit anti-CD45 (Abcam, ab10558; 1:1000) and rabbit anti-GAPDH (Cell Signaling Technology, CST-5174; 1:2000). The membranes were then incubated with horseradish peroxidase-conjugated secondary antibodies (Koma Biotech, Seoul, Korea), and protein bands were visualized using enhanced chemiluminescence (ECL) reagents (Daeil Lab Inc., Seoul, Korea). Glyceraldehyde 3-phosphate dehydrogenase (GAPDH) was used as a loading control. Band intensities were analyzed using Image J.

#### Immunohistochemistry

For immunofluorescence staining, sections were washed with PBS containing 0.1% Triton X-100 (PBST), treated with 1% BSA, and incubated with combinations of primary antibodies: rat anti-CD45 antibodies (MyBioSource, cat. no. MBS520149; 1:1000), chicken anti-GFAP antibodies (Synaptic Systems, cat. no. 173006; 1:1000), mouse anti-M-CSF antibodies (R&D Systems, cat. no. AF416; 1:500), Goat anti-ARG antibodies (Abcam, cat. no. ab60176; 1:500), Guinea pig anti-MAP2 antibodies (Synaptic Systems, cat. no. 188004; 1:200), mouse anti-PSD95 antibodies (Thermo Fisher Scientific, cat. no. MA1-045; 1:100), and chicken anti-VGLUT1 antibodies (Synaptic Systems, cat. no. 135309; 1:500). Immunoreactive proteins were visualized with appropriate fluorescence-conjugated secondary antibodies (1:500 dilution; Invitrogen, Carlsbad, CA). Sections were counterstained with 4',6-diamidino-2-phenylindole (DAPI; Sigma) to detect nuclei. Images were captured using a confocal microscope (Carl Zeiss 800, Oberkochen, Germany).

#### Statistical analysis

Statistical analysis was performed using GraphPad Prism software (Prism, San Diego, CA). Data were analyzed using a two-tailed unpaired t-test for comparisons between two groups. One-way or two-way analysis of variance (ANOVA) was applied to compare multiple groups. All values are means ± SEM of at least three independent experiments; "n" values represent the number of tissue samples, or animals analyzed per group.

## Results

### Diabetes alters gene expression in the brain

To investigate the mechanisms by which diabetes aggravates initial brain injury and delays subsequent repair, diabetes was induced by intraperitoneal (i.p.) injection of streptozotocin (STZ), and focal brain injury was produced by stereotaxic injection of ATP into the striatum. STZ is widely used to model type 1 diabetes by inducing pancreatic β-cell destruction and sustained hyperglycemia, which in turn promotes chronic neuroinflammatory and neurodegenerative processes [36–38]. ATP, a well-characterized component of damage-associated molecular patterns (DAMPs), was used to induce quantifiable brain injury [31, 34]. The striatum was selected as the target site because it is critically involved in neurodegenerative diseases such as Parkinson’s disease and Huntington’s disease [39, 40].

**Fig. 1:**
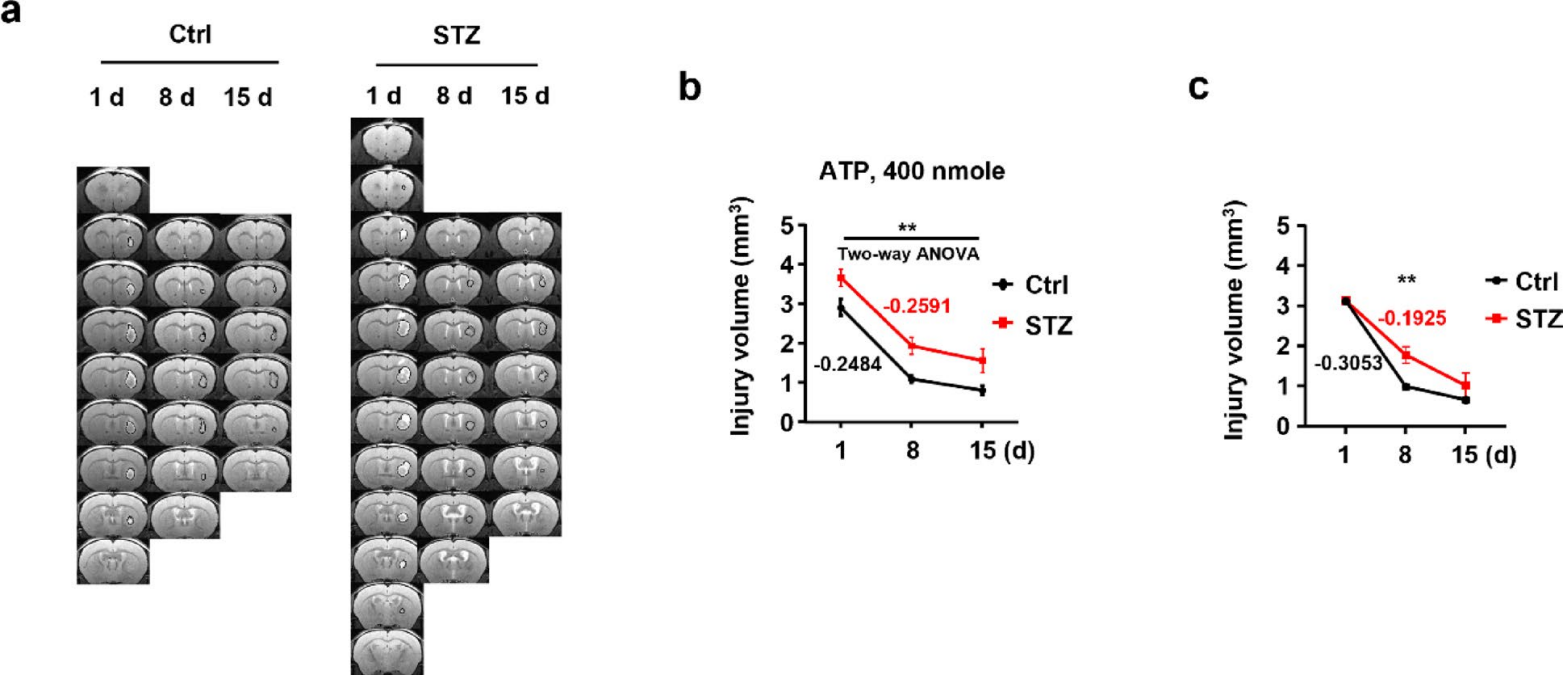
STZ increases ATP-induced brain injury and delays repair. **a** Hyperglycemia was induced by intraperitoneal (i.p.) injection of low-dose streptozotocin (STZ; 50 mg/kg) for five consecutive days. Focal brain injury was produced by stereotaxic injection of ATP (400 nmol) into the striatum of control and STZ-treated mice. Brain injury was analyzed in mice anesthetized with isoflurane at the indicated times after ATP injection using 9.4 T MRI. T2-weighted 2D Turbo RARE sequences were acquired using the following parameters: TR/TE, 9,000/33 ms; resolution, 78 × 78 × 250 μm^3^; slice thickness, 250 μm; RARE factor, 8; average, 2; scan time, 9 min 36 s. **b** Lesion volumes were quantified from MR images using ImageJ. **(c)** Time-dependent changes in lesion volume in control and STZ mice with similar initial lesion sizes (~ 3 mm^3^). Data are presented as mean ± SEM from at least three mice. Statistical significance was determined by two-way ANOVA (b) and one-way ANOVA (c). **p < 0.005

Magnetic resonance imaging (9.4 T MRI) revealed that in STZ-treated mice, the initial lesion size was larger compared to controls. By 15 days post-injury, the damage size was reduced in both groups with a comparable slope of reduction (Fig. 1a, b). However, the slope of recovery was steeper when the initial lesion size was larger (SFig. 2). Therefore, we compared lesion repair between groups with similar initial lesion volumes (approximately 3 mm^3^), and found that under these conditions, lesion repair was delayed in STZ-treated mice (Fig. 1c).

**Fig. 2:**
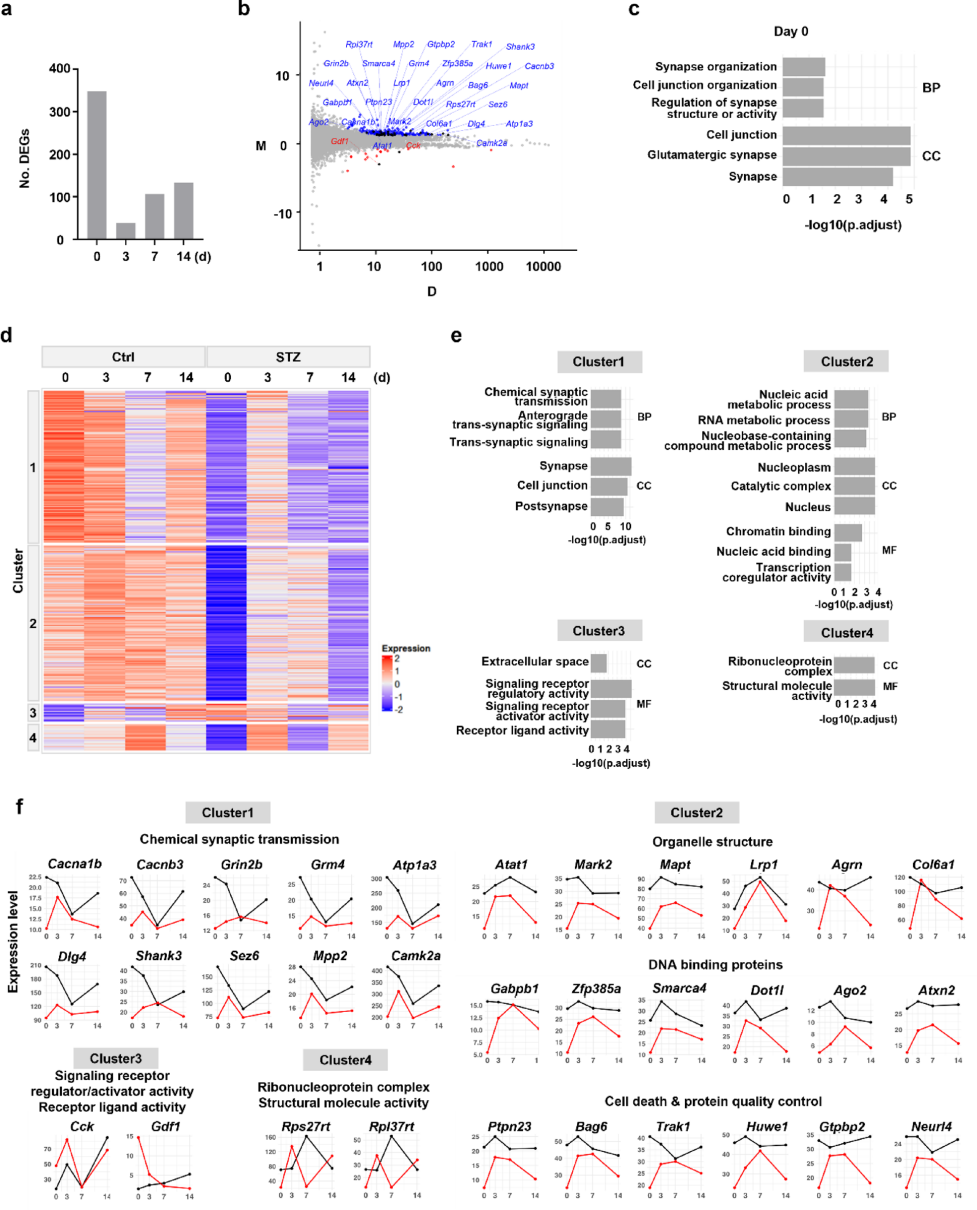
STZ altered brain transcriptomic response to brain injury. **a** Differential expression across days 0, 3, 7, and 14 after injury; day 0 showed the greatest number of DEGs (348 genes). **b** MD (Mean-Difference) plot for day 0 comparing STZ to control. M values represent log fold changes, and D values represent log-scaled absolute expression differences. Points are colored by DEG status: non-DEGs (gray), genes upregulated in STZ (red), and genes downregulated in STZ (blue); thresholds for M and q follow the Methods. **c** Gene Ontology (GO) enrichment for day-0 DEGs (top terms shown; FDR-adjusted P-values). **d** K-means clustering heatmap of day-0 DEGs across samples; rows are z-scored, with relative up-regulation in red and down-regulation in blue (STZ vs control). **e** GO enrichment for each cluster identified in panel D. **f** Time-course expression profiles highlighting genes within selected GO terms that show marked group differences between control and STZ

To investigate gene expression dynamics changed by STZ, we performed RNA-seq at days 0, 3, 7, and 14 post-injury. Interestingly, the highest number of differentially expressed genes (DEGs) was observed at day 0, prior to injury (Fig. 2a). We first examined genes whose expression was altered by diabetes alone (day 0). Compared to controls, the striatum of STZ-treated mice exhibited 18 upregulated and 330 downregulated genes (Fig. 2b). These genes are associated with GO terms such as ‘synapse organization’ and ‘cell junction’ (Fig. 2c). Next, we categorized these genes into four clusters based on their post-injury expression patterns and performed GO term analysis for each cluster (Fig. 2d). Cluster 1 genes, which show the greatest reduction at day 7 after brain injury in controls and are already downregulated by diabetes even before injury, are enriched in synapse-related GO terms, particularly those involved in ‘synaptic transmission/signaling' (Fig. 2e, f). Notably, many of these genes are linked to excitatory synapses (Fig. 2f). We further evaluated the expression of VGLUT1 and PSD95 using immunostaining, as these proteins are well-established excitatory pre- and post-synaptic markers, respectively. Compared with the control brains, PSD95 intensity in STZ brains showed a decreasing trend at 3 days, while VGLUT1 intensity showed a decreasing trend at intact, 3 days, and 14 days post-injury; however, these differences did not reach statistical significance (SFig. 3).

Cluster 2 genes, enriched for organelle organization–related GO terms, are unaffected by brain injury in controls but show decreased expression in response to diabetes alone and to brain injury after 14 days in diabetic animals (Fig. 2e, f). Notably, several microtubule-related genes were altered in STZ-treated animals, including *Atat1* (tubulin N-acetyltransferase), *Mark2* (MAP/microtubule affinity–regulating kinase 2), *Mapt* (microtubule-associated protein tau), and *Lrp1* (LDL receptor–related protein 1, a receptor for tau proteins). Other cytoskeleton-related genes were also affected, such as *Agrn* (a component of the basal lamina) and *Col6a1* (a constituent of type VI collagen). In addition, Cluster 2 contains DNA-binding protein–encoding genes involved in transcription and chromatin remodeling, as well as genes associated with cell death and protein quality control (*Ptpn23, Bag6, Trak1, Huwe1, Gtpbp2,* and *Neurl4*). Clusters 3 and 4 contain relatively few genes, with only a subset assigned to GO terms such as signaling and ribonucleoproteins (Fig. 2e, f). Overall, these findings suggest that diabetes affects synaptic organization and cell death pathways in intact brains.

### STZ alters gene expression in PBMCs

**Fig. 3:**
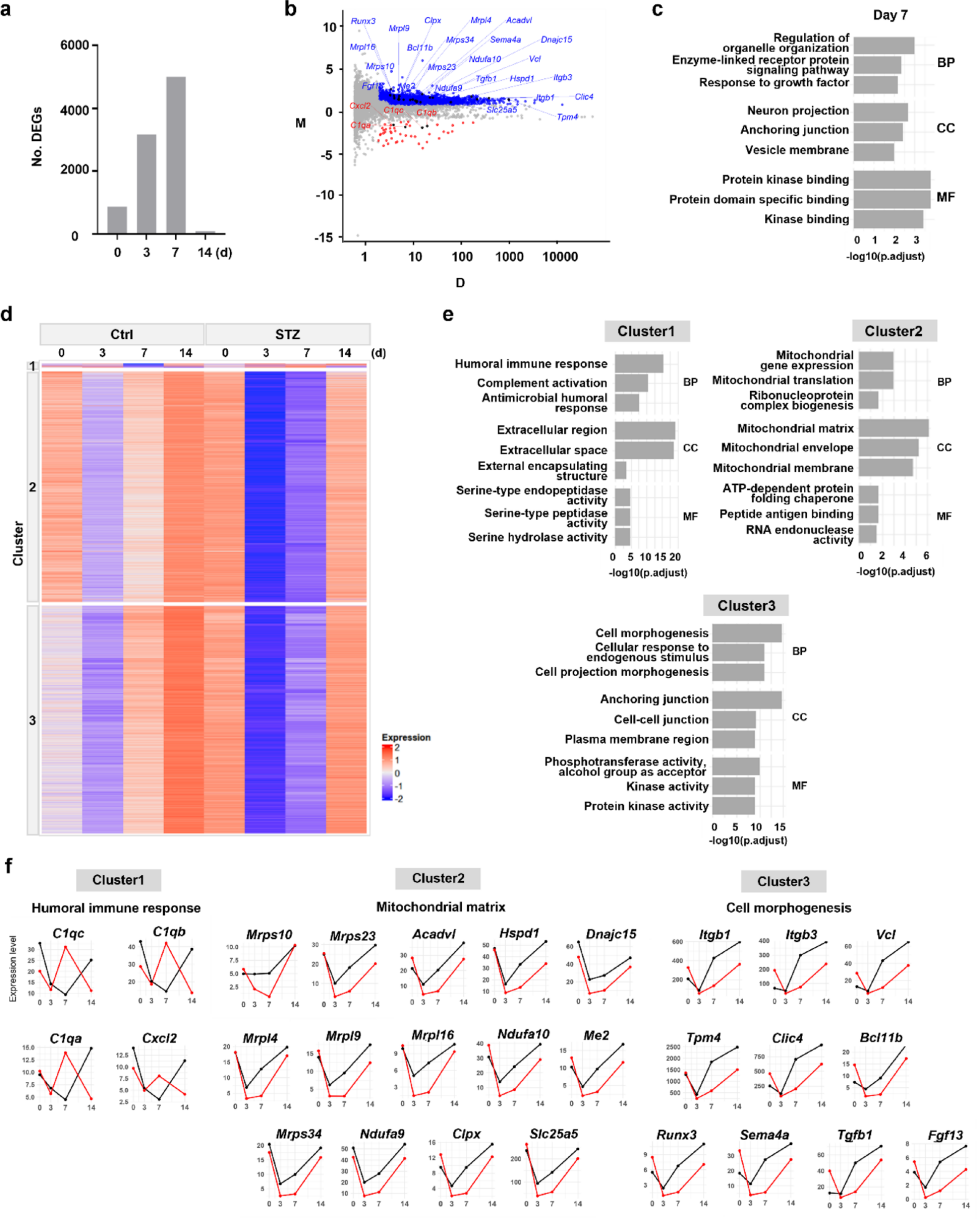
STZ altered PBMCs transcriptomic response to brain injury. **a** Differential expression across days 0, 3, 7, and 14 in PBMCs; day 7 shows the greatest number of DEGs (5,000 genes). **b** MD plot for day 7 comparing STZ to control. Points are colored by DEG status: non-DEGs (gray), genes upregulated in STZ (red), and genes downregulated in STZ (blue); thresholds for M and q are defined in the Methods. **c** Gene Ontology (GO) enrichment for day-7 DEGs (FDR-adjusted P values; top terms shown). **d** K-means clustering heatmap of day-7 DEGs across samples; rows are z-scored, with relative up-regulation in STZ (red) and down-regulation (blue) versus control. **e** GO enrichment for each cluster identified in panel D. **f** Time-course expression profiles highlighting genes within selected GO terms that show marked group differences between STZ and control

We examined how STZ alters the properties of circulating monocytes, as these cells infiltrate the injured brain tissue and critically influence post-injury repair processes [41, 42]. Transcriptomic profiling of peripheral blood mononuclear cells (PBMCs) revealed that the number of differentially expressed genes (DEGs) peaked at 7 days after brain injury (Fig. 3a). Compared with controls, STZ-treated animals exhibited 46 upregulated genes and 4,954 downregulated genes (Fig. 3b). Gene Ontology (GO) analysis indicated that these DEGs were broadly associated with protein binding and structural organization (Fig. 3c). Because these GO categories were overly general, we further stratified the DEGs based on their temporal expression patterns before and after brain injury. DEGs were clustered into three distinct groups (Fig. 3d), and GO analysis was subsequently re-performed for each cluster to identify time-dependent functional changes (Fig. 3e).

By 7 days post-injury, control mice exhibited downregulation of humoral and innate immune transcripts, including complement components *C1qa–c* and the chemokine receptor *Cxcr2* (Cluster 1; Fig. 3d–f). In contrast, STZ-treated mice showed aberrant upregulation of these genes. Because complement signaling and CXCR2 are known to mediate synapse elimination and myeloid cell recruitment [43, 44], these changes suggest exacerbated inflammatory responses and a likely impairment of tissue remodeling and functional recovery in injured STZ brains.

Cluster 2 genes were downregulated by brain injury and further suppressed in the diabetic condition. Gene Ontology analysis revealed strong enrichment for mitochondrial-related processes. The repressed transcripts encompassed mitochondrial translation (e.g., *Mrps10, Mrps23, Mrps34, Mrpl4, Mrpl9,* and *Mrpl16*), electron transport and oxidative phosphorylation (*Ndufa9, Ndufa10*), fatty-acid β-oxidation (*Acadvl*), tricarboxylic acid and auxiliary metabolism (*Me2*), chaperone-mediated proteostasis (*Hspd1, Clpx, Dnajc15*), metabolite transport (*Slc25a5*), and mitophagy or mitochondrial quality control (*Nipsnap1*). Collectively, this transcriptional signature is consistent with reduced oxidative phosphorylation capacity and impaired mitochondrial proteostasis in circulating immune cells, a phenotype previously associated with dysfunctional immune responses in metabolic disease states [45, 46].

### STZ alters infiltrated monocyte distribution and attenuates astrocyte activation

**Fig. 4.**
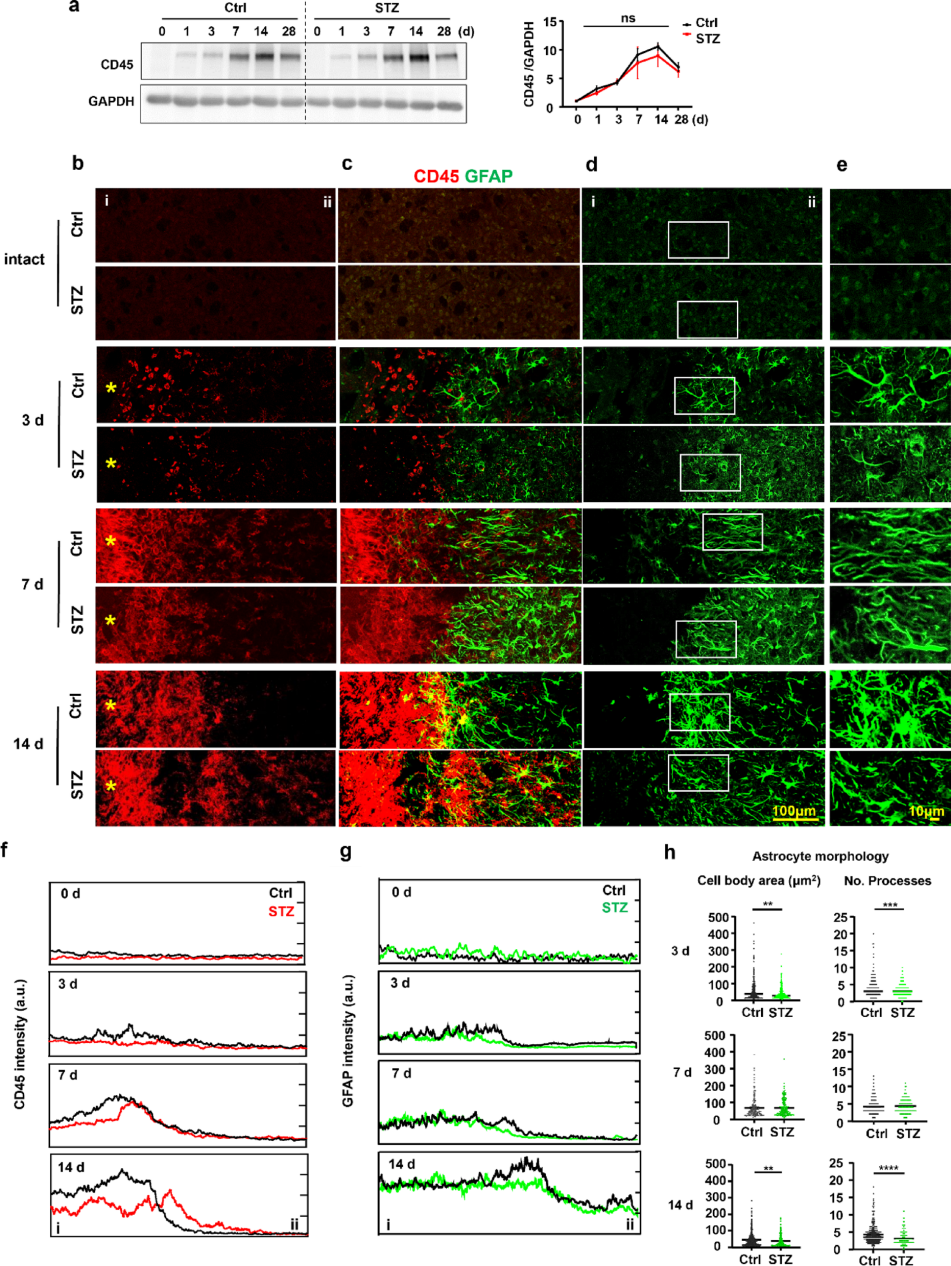
Monocyte infiltration and spatial distribution in injured STZ brains. **a** Monocyte infiltration into the injured brains was assessed by western blotting for CD45. GAPDH, a loading control. Band intensities of CD45 were normalized to GAPDH and quantified (right). Values are mean ± SEM (n = 3). Statistical significance was determined by one-way ANOVA; ns, not significant. **b**–**e** Brain sections of injured brains were immunostained for CD45 (red) and GFAP (green). **f**–**g** Relative fluorescence intensity of CD45 (f) and GFAP (g) was quantified across a linear region from the lesion core (i) to the perilesional region (ii) using ImageJ. **h** The size of cell bodies and the number of processes of astrocytes were measured with MetaMorph software. Statistical significance was determined using the Mann–Whitney test (**p < 0.01, ***p < 0.001, ****p < 0.0001)

Next, we examined monocyte infiltration into the injured brains of control and STZ mice. Western blot analysis of whole-brain lysates showed that CD45, a marker of infiltrating monocytes, became detectable within 1 day post-injury, peaked at 14 days, and declined by 28 days in control and STZ mice without significant differences between the two groups across time points (Fig. 4a). Interestingly, however, immunohistochemical analysis revealed that STZ changed monocyte distribution, particularly at 14 days post-injury (Fig. 4b, f). In STZ brains compared to control brains, monocytes showed a dispersed distribution, resulting in a lower monocyte density in the injury core (Fig. 4b, f). These findings indicate that although overall CD45 abundance is largely unchanged, STZ treatment alters the spatial pattern and extent of monocyte infiltration in the injured brain.

It has been reported that astrocytes form barriers around lesions to confine monocytes [41–43]. Here, we analyzed astrocytes with GFAP antibodies and found that astrocyte activation was attenuated in the STZ-treated brains (Fig. 4d, e, g, h). In intact WT and STZ brains, the intensity of GFAP was similarly low, but at 3 and 14 days post-injury, astrocyte hypertrophy and process formation were significantly reduced in the STZ mice (Fig. 4d, e, h). Particularly, at 14 d, astrocyte barrier was not properly formed (Fig. 4d, e). These results suggest that attenuated astrocyte barrier formation likely contributes to dispersed monocyte localization in diabetic brains.

### STZ alters transcriptomic profile of infiltrated monocytes

**Fig. 5:**
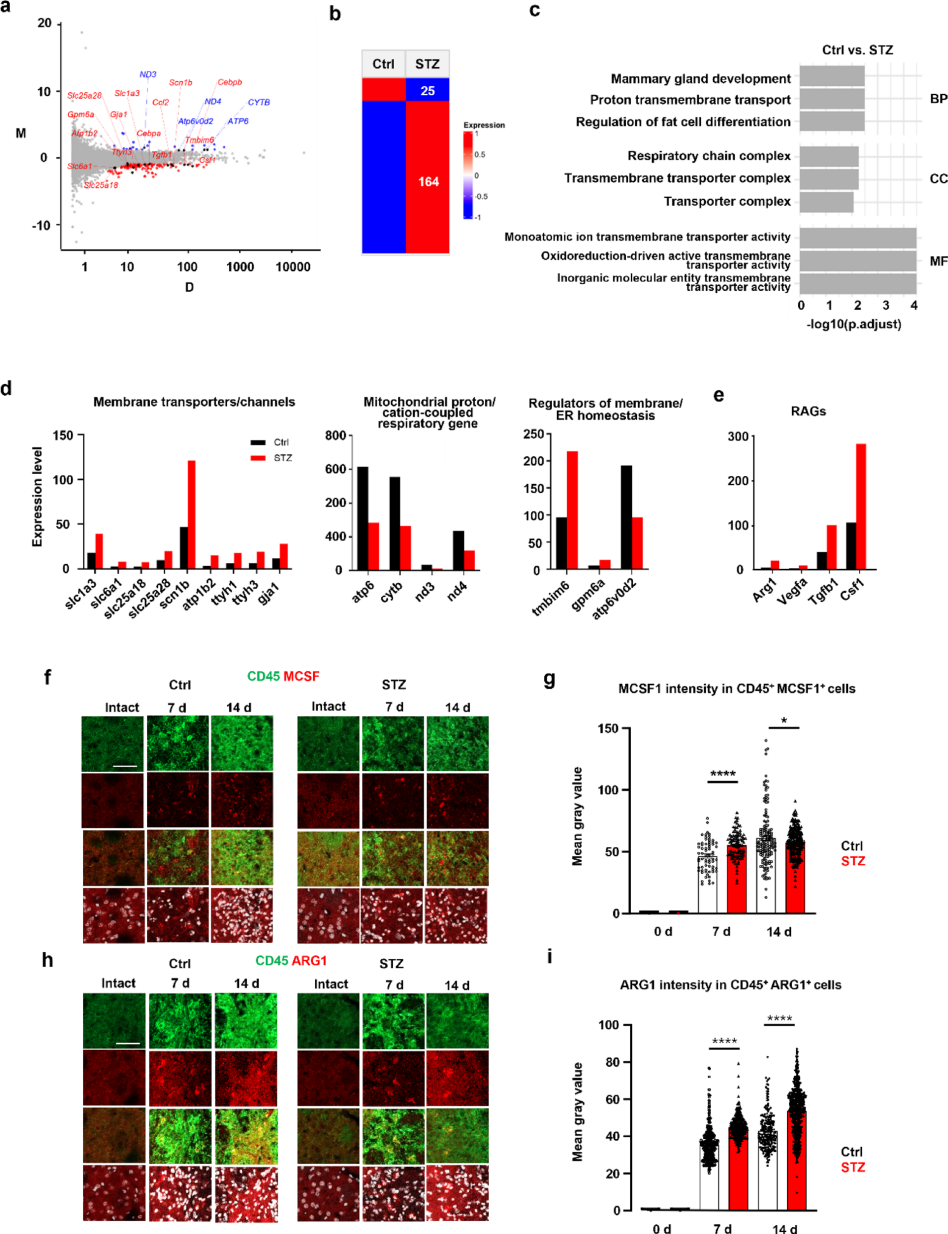
STZ altered gene expression in infiltrated monocytes into the injured brains. **a** Differential expression of genes in infiltrating monocytes at 7 days post-injury identified 164 genes upregulated and 25 down-regulated in STZ versus control. **b** Heatmap of DEGs across samples; rows are z-scored, with relative up-regulation in STZ (red) and down-regulation (blue) versus control. **c** Gene Ontology (GO) enrichment for 7 d DEGs (FDR-adjusted P values; top terms shown). **d** Bar plots of representative genes within selected GO terms highlighting expression differences between control (black) and STZ (red). **e** RAGs in monocytes in control (black) and STZ (red) at day 7. **f**, **h** Representative immunofluorescence images of the penumbra region in the injured striatum stained for CD45/MCSF1 (f) and CD45/ARG1 (h). Scale bars = 50 μm. **g**, **i** MCSF1 levels (g) and ARG1 levels (i) merged with CD45 were quantified using ImageJ. Each dot represents the intensity of MCSF1 or ARG1 in an individual cell. Values are mean ± SEM (n ≥ 3 mice per group). * *p* < 0.05, **** *p* < 0.0001 vs. Ctrl by Student's t-test

Next, we performed RNA sequencing at 7 days post-injury to define diabetes-induced transcriptional changes in infiltrating monocytes. STZ treatment resulted in the upregulation of 164 genes and downregulation of 25 genes relative to controls (Fig. 5a, b). Gene Ontology analysis revealed STZ upregulated genes related to ion-handling and membrane-associated pathways, including solute carriers and ion channels (*Slc1a3, Slc6a1, Slc25a18, Slc25a28, Scn1b, Atp1b2, Ttyh1, Ttyh3,* and *Gja1*), and regulators of membrane and endoplasmic reticulum homeostasis (*Tmbim6, Gpm6a,* and *Atp6v0d2*) (Fig. 5c, d). However, STZ reduced mitochondrial respiratory genes encoded by the mitochondrial genome (*mt-Atp6, mt-Cytb, mt-Nd3, mt-Nd4,* and *mt-Nd4l*) (Fig. 5c, d). Notably, repair-related genes (RAGs, *Tgfb1, Arg1, Vegfa,* and *Csf1*) were paradoxically upregulated (Fig. 5e). To validate these transcriptomic findings at the protein level, we analyzed the levels of MCSF1 (CSF1) and ARG1 in CD45( +)-infiltrated monocytes using immunostaining. The levels of MCSF1 and ARG1, particularly those merged with CD45, were significantly increased at 7 and 14 days post-injury. Collectively, these results suggest that diabetes induces coordinated remodeling of membrane transport and ion-channel programs in infiltrating monocytes while suppressing pathways.

## Discussion

Our study demonstrates that diabetes profoundly alters both the brain and peripheral immune system, predisposing the brain to more severe damage and impaired repair following injury. Previous studies have reported synaptic dysfunction and cognitive decline in diabetic models [44–46], and our data provide transcriptomic evidence linking diabetes to impaired synaptic architecture. In response to injury, excitatory synaptic transmission and signaling genes were downregulated in controls, but in STZ brains, these genes were suppressed even before injury. In the brain, diabetes reduced expression of genes related to microtubule- and cytoskeleton-associated pathways (*Atat1, Mark2, Mapt, Lrp1, Agrn, Col6a1*) as well as cell death and protein quality control genes (*Ptpn23, Bag6, Trak1, Huwe1, Gtpbp2, Neurl4*). Disruption of these pathways is known to impair axonal regeneration, destabilize synaptic architecture, and increase neuronal susceptibility to degeneration [47–52].

The impact of diabetes on the brain injury indicates active bidirectional communication between the brain and peripheral immune system. These findings suggest that diabetes and brain injury together induce a broad metabolic and structural remodeling of circulating immune cells. Consistent with this, the behavior of infiltrating monocytes was profoundly altered in diabetic mice. Monocyte recruitment to the lesion showed little change, but these cells displayed a scattered distribution in the injured parenchyma. RNA-seq analysis revealed infiltrated monocytes in STZ mice upregulated ion transporter and channel genes, including *Slc* and *Ttyh* family members, while downregulating mitochondrial respiratory genes. Given the critical role of monocyte-derived macrophages in debris clearance and tissue remodeling [3, 53], such dysfunction is likely to compromise repair processes and contribute to the poorer injury outcomes observed under diabetic conditions. Unexpectedly, diabetic monocytes exhibited increased expression of RAGs, including *Tgfb1* and *Csf1*, despite delayed tissue repair. This paradoxical state suggests that diabetes drives infiltrating monocytes into a metabolically constrained and functionally altered phenotype, in which ionic imbalance and reduced bioenergetic capacity may override reparative transcriptional programs, ultimately limiting their ability to support tissue recovery.

Monocyte infiltration into the injured brain is governed by multiple factors, including the strength of chemotactic cues, cellular motility, and the structural properties of the vasculature. Previous studies have shown that STZ treatment reduces vessel diameter and density in the intact brain [54, 55], potentially limiting physical entry routes for circulating leukocytes. Transcriptomic analyses revealed slightly reduced expression of the chemokine, *Cx3cl1,* in the brain (SFig. 4a), and a chemokine receptor, Ccr2 in PBMCs (SFig. 4b), and Cx3cr1 in infiltrating monocytes (SFig. 4c). However, diabetes did not significantly affect the amount of infiltrated monocytes in response to brain injury.

Impaired astrocyte activation in diabetic brains may contribute to attenuated chemokine expression, and defects in astrocyte barrier formation, resulting in dispersed monocyte distribution. Such defects in astrocyte–immune communication likely contribute to uncoordinated repair processes and delayed recovery. Future studies should investigate how diabetes reshapes astrocyte transcriptomes to elucidate the molecular basis of their altered function.

These findings provide mechanistic insight into the long-recognized but poorly understood clinical observation that diabetic patients experience poorer outcomes after brain injury. Our study suggests synaptic, cytoskeletal, and mitochondrial programs as convergent points of vulnerability. Targeting astrocyte activation or restoring immune cell function could help re-establish effective neuroimmune communication and improve repair capacity in the diabetic brain.

## Supplementary Information

Below is the link to the electronic supplementary material.

Supplementary Material 1.

## Data Availability

The datasets used and/or analyzed during the current study are available from the corresponding author on reasonable request.
