# Supplementary material for: Diabetes reprograms brain and systemic immune landscapes to impair repair after injury
Source: Mol Brain. 2026 Jun 9;19:44. doi: 10.1186/s13041-026-01310-5 (PMC13251158; doi:10.1186/s13041-026-01310-5)
Supplement: Supplementary file 1 — Supplementary Material 1. [file 13041_2026_1310_MOESM1_ESM.docx]

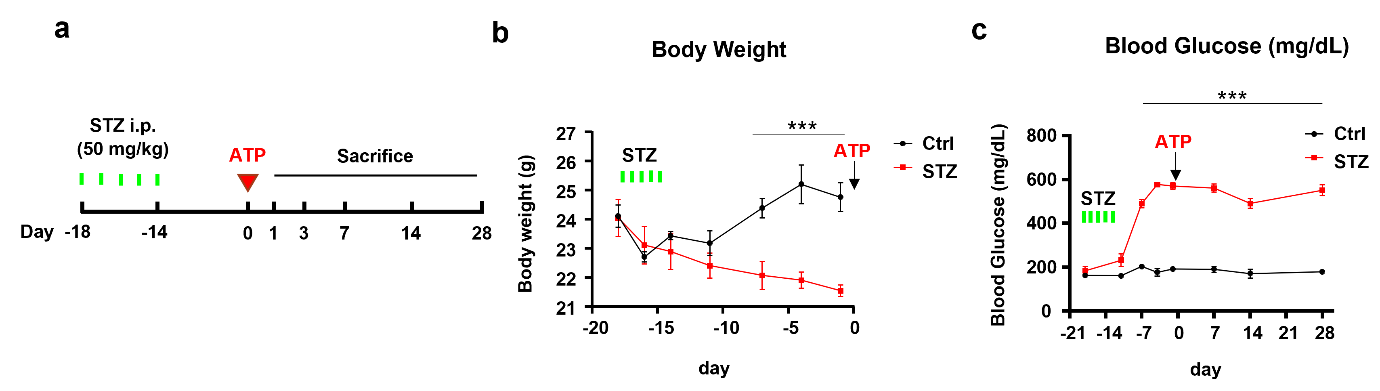


**SFigure 1. STZ increases ATP-induced brain injury and delays repair.**
**(a)** Hyperglycemia was induced by intraperitoneal (i.p.) injection of low-dose streptozotocin (STZ; 50 mg/kg) for five consecutive days. Focal brain injury was produced by stereotaxic injection of ATP (400 nmol) into the striatum of control and STZ-treated mice.

**(b)** Body weight was monitored at the indicated time points.

**(c)** Blood glucose levels were monitored at the indicated time points. Data are presented as mean ± SEM from at least three mice. Statistical significance was determined by one-way ANOVA. ***p < 0.001.


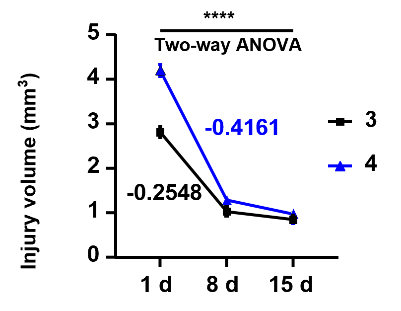


**SFigure 2. A larger initial injury size was associated with a steeper slope of recovery.**

Data are presented as mean ± SEM from at least three mice. Statistical significance was determined by two-way ANOVA. ****p < 0.0001.


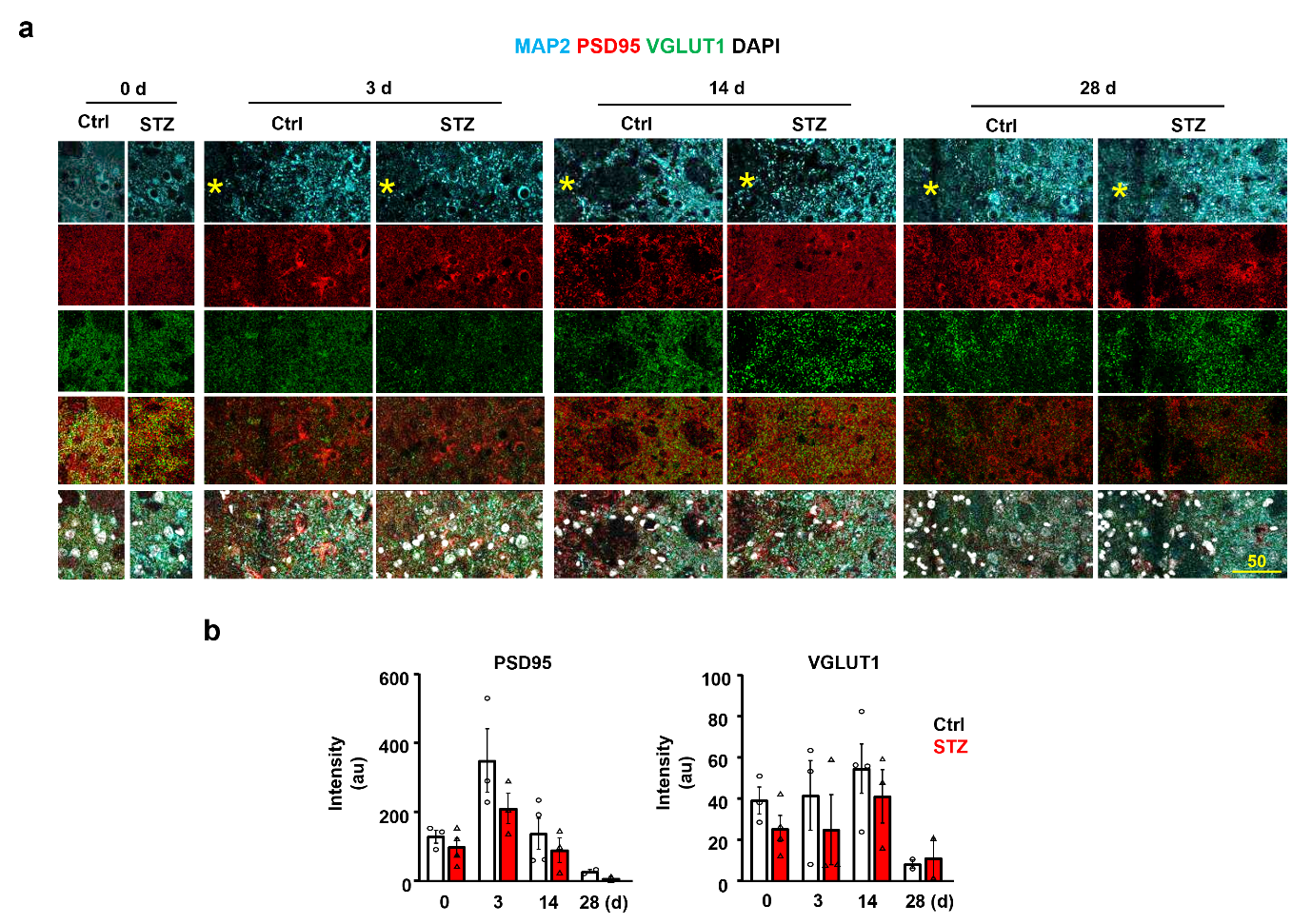


**SFigure 3. STZ delays the recovery of excitatory synaptic proteins, PSD95 and VGLUT1, in injured brains.**

**(a)** Intact and injured brain sections obtained at the indicated time points from control and STZ mice were stained for PSD95 (red), VGLUT1 (green), and MAP2 (cyan). *, injury core.

**(b)** The intensities of PSD95 and VGLUT1 across the entire area of the images shown in (a) were measured using LAS X software. x-axis: 0 = total darkness, and 255 = maximum brightness; y-axis: the number of pixels at each intensity. Data are presented as mean ± SEM (n ≥ 3 mice per group).


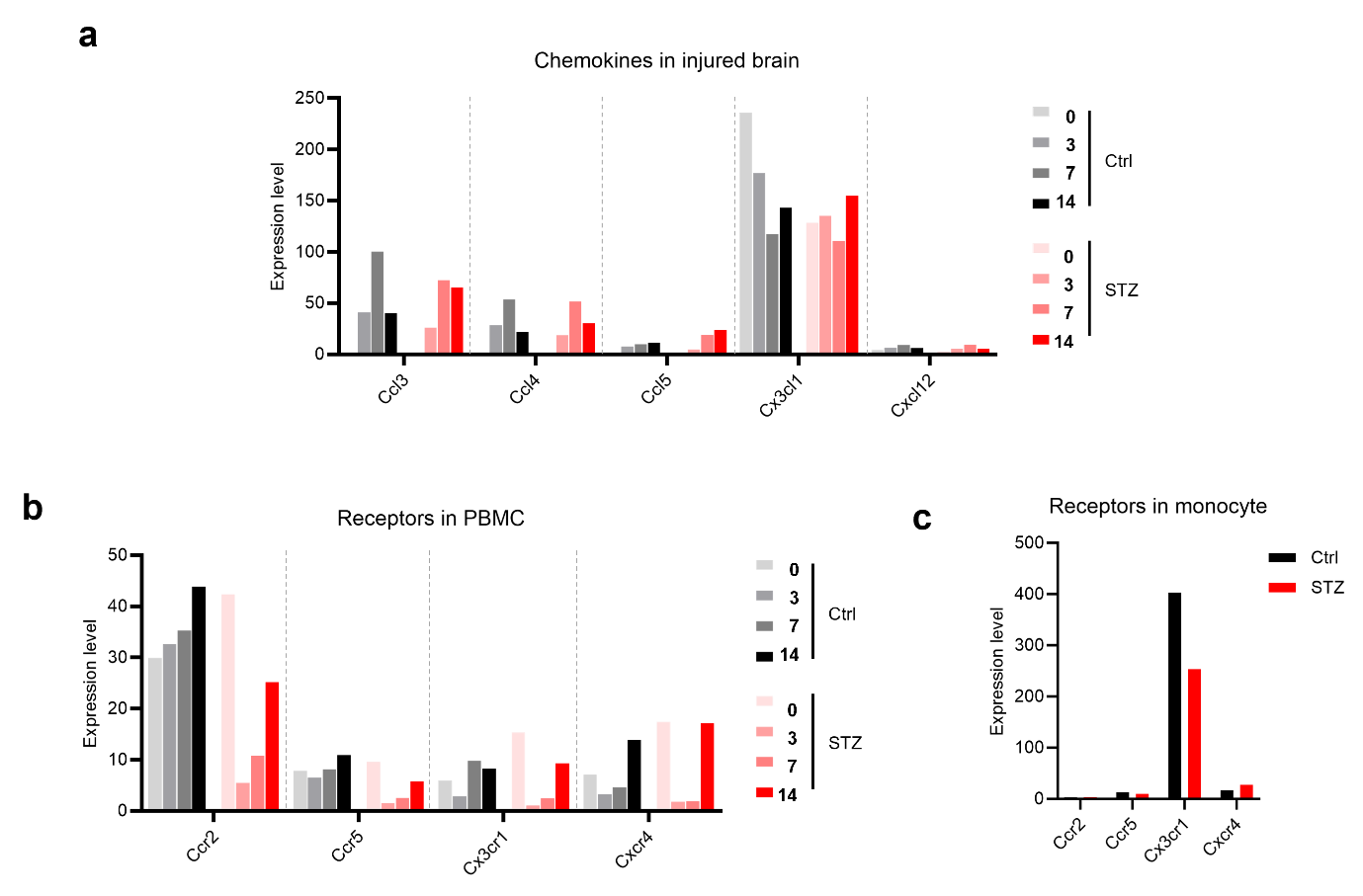


**SFigure 4. Expression** **of chemokines and chemokine receptors** **in the brains, PBMCs, and monocytes in the control and STZ mice**

**(a)** Expression of chemokines in injured brains of control and STZ mice.

**(b)** Expression of chemokine receptors in PBMCs.

**(c)** Expression of chemokine receptors in infiltrated monocytes in injured brains at 7 d.
